# Early postmortem beef muscle proteome and metabolome variations due to supranutritional zinc and ractopamine hydrochloride supplementation

**DOI:** 10.1093/jas/skae272

**Published:** 2024-09-16

**Authors:** Matthew D Schulte, Katherine G Hochmuth, Edward M Steadham, Steven M Lonergan, Stephanie L Hansen, Elisabeth Huff-Lonergan

**Affiliations:** Department of Animal Science, Iowa State University, Ames, Iowa 50011, USA; Department of Animal Science, Iowa State University, Ames, Iowa 50011, USA; Department of Animal Science, Iowa State University, Ames, Iowa 50011, USA; Department of Animal Science, Iowa State University, Ames, Iowa 50011, USA; Department of Animal Science, Iowa State University, Ames, Iowa 50011, USA; Department of Animal Science, Iowa State University, Ames, Iowa 50011, USA

**Keywords:** muscle metabolome, muscle proteome, pH decline, ractopamine hydrochloride, tenderness, zinc

## Abstract

It was hypothesized that the longissimus thoracis (**LT**) muscle proteome, phosphoproteome, and metabolome could explain postmortem metabolism and tenderness differences in muscle from cattle supplemented zinc (**Zn**) and/or ractopamine hydrochloride (**RH**). High percentage Angus steers (*N* = 20) were fed in a 2 × 2 factorial assigned to Zn and RH treatments: control (CON; *n* = 10; analyzed 36 mg Zn/kg dry matter [**DM**]) or supranutritional Zn supplementation (SUPZN; *n* = 10; control diet + 60 mg Zn/kg DM [from ZnSO_4_] + 60 mg Zn/kg DM [from Zn–amino acid complex]) for the entire 89-d trial. During the 28 d before harvest, steers were blocked by body weight within Zn treatments to RH treatments of 0 (NO; *n* = 10) or 300 mg (RAC; *n* = 10) per steer per day. Steers were harvested at the Iowa State Meat Laboratory, where pH decline (1, 3, 6, and 24 h postmortem) was measured. At 24 h postmortem, LT muscle sections were removed from carcasses, and steaks were analyzed for Warner–Bratzler shear force (WBSF) values at 1, 3, 7, and 14 d postmortem. Muscle samples were taken at 1 h, 1, 3, 7, and 14 d postmortem for the following analysis: troponin-T degradation (1, 3, 7, and 14 d postmortem), myosin heavy chain analysis (1 h postmortem), sarcoplasmic proteome analysis through tandem mass tagging analysis (1 h and 1 d postmortem), metabolome analysis (1 h and 1 d postmortem), and phosphoproteome analysis (1 h postmortem). SUPZN-NO tended to have a lower (*P* = 0.06) pH at 6 h postmortem and a lower WBSF value (*P* = 0.06) at 1 d postmortem. CON-RAC had a higher (*P* = 0.04) pH at 6 h postmortem and WBSF value (*P* < 0.01) at 1 d postmortem. A lower pH at 6 h postmortem and lower WBSF value at 1 d postmortem in the SUPZN-NO treatment was accompanied by more sorbitol and fructose at 1 d postmortem, and less myosin regulatory light chain 2 at 1 h postmortem, and less adenosine monophosphate deaminase 1 (AMPD1) at 1 d postmortem than all other treatments. A higher pH at 6 h postmortem and higher WBSF value at 1 d postmortem in CON-RAC and SUPZN-RAC was accompanied by more soluble structural proteins (troponin-T and myosin-7) at 1 h postmortem than CON-NO. At 1 h postmortem, CON-RAC had more glyceraldehyde-3-phosphate dehydrogenase than CON-NO or SUPZN-RAC. Differences in energy metabolism enzymes, metabolites, and structural proteins may affect ATP production, rigor development, and lactate buildup which may explain the differences in postmortem metabolism and tenderness development at 1 d postmortem.

## Teaser

Beef tenderness can be driven by management practices that affect muscle growth. Altering muscle growth influences proteomic and metabolomic features that affect beef quality.

## Introduction

Optimization of growth and efficiency is essential to improve the beef industry’s sustainability. Among the ways to optimize growth efficiency are the use of growth-promoting technologies, such as ractopamine hydrochloride (**RH**), and advanced management practices, such as supplementing zinc (**Zn**) above the National Academy of Sciences, Engineering, and Medicine (Nutrient Requirements of Beef Cattle, 8th rev. ed., 2016) recommendations. An example of supranutritional Zn supplementation is feeding Zn at 5 times the recommendation (SUPZN, 150 mg Zn/kg dry matter [**DM**] [Genther-Schroeder et al., 2018]). RH supplementation improves protein accretion in muscle due to less protein degradation (Mersmann, 1998), increased cattle growth efficiency, and increased carcass gains (Lean et al., 2014). A synergistic effect of RH supplementation with Zn–amino acid supplementation has been demonstrated in beef cattle (Genther-Schroeder et al., 2016). However, as improvements are made in growth and carcass characteristics, the industry must ensure negative impacts on beef quality, and tenderness are not introduced.

Supplementation of RH consistently results in negative but minimal impacts on beef tenderness during the first 14 d postmortem (Lean et al., 2014). The influence of Zn supplementation has resulted in mixed results on beef tenderness (Bohrer et al., 2014; Edenburn et al., 2016; Vellini et al., 2020; Schulte et al., 2021). Improvements in early (1 d postmortem) Warner–Bratzler shear force (**WBSF**) values in Angus steers (Schulte et al., 2021) were identified when fed 150 mg Zn/kg DM (basal diet Zn [36 mg Zn/kg] + 60 mg Zn/kg from Zn–amino acid complex + 60 mg Zn/kg from ZnSO_4_). Zn has been noted to have an impact after aging as well (Vellini et al., 2020). Improvements in WBSF values at 28 d postmortem have been observed when Zn amino acid complex (3.11 kg) or Zn–amino acid with chromium (2.86 kg) were fed in place of inorganic sources of Zn (3.45 kg) to Nellore bulls (Vellini et al., 2020). Others have demonstrated no difference in WBSF values from cattle supplemented Zn. In crossbred steers fed a control diet, RH only (400 mg per head per day; 36.98 mg Zn/kg DM), RH + 1 g Zn-propionate per head per day (97 mg Zn/kg DM), RH + 3 mg of chromium-propionate (39 mg Zn/kg DM), or the combination of all 3 diets (101 mg Zn/kg DM) were not different in WBSF values (3.62, 3.86, 3.61, and 3.40 kg, respectively) at 14 d postmortem (Edenburn et al., 2016). In a similar study (Bohrer et al., 2014), crossbred Angus steers were fed one of 3 treatments: control (50 mg Zn/kg DM), RH only, and RH + trace minerals (1 g of Zn as Zn-propionate [160 mg Zn/kg DM] and 3 mg of chromium-propionate). In the study by Bohrer et al. (2014), WBSF values were analyzed on steaks at 14 d postmortem, and no difference between treatments (control = 2.70 kg; RH only = 3.01 kg; combination = 3.16 kg) was identified. The source, days on treatment, and concentration fed could explain these differences in WBSF value due to Zn supplementation.

Antemortem factors such as feeding RH and supranutritional Zn affect postmortem bovine muscle metabolism. Those practices impact tenderness development through early postmortem pH decline and postmortem protein degradation (Schulte et al., 2021). Postmortem pH decline can dictate WBSF values (Marsh et al., 1987; Hwang and Thompson, 2001). Marsh et al. (1987) demonstrated that an intermediate pH decline (pH 5.8 to 6.4 at 3 h postmortem) resulted in more tender steaks at 14 d postmortem, while a slow pH decline (pH > 6.4 at 3 h postmortem) resulted in the least tender steaks. Schulte et al. (2021) reported that RH-only supplementation resulted (*P* < 0.01) in the toughest steaks at 1 d postmortem, while meat from cattle supplemented supranutritional Zn-only tended to have a lower WBSF value (*P* = 0.06) at 1 d postmortem. This difference in WBSF values between treatments was explained by a trend (*P* = 0.06) for lower pH values at 6 h postmortem in supranutritional Zn-only supplementation, and greater pH values (*P* = 0.04) in cattle supplemented RH at 6 h postmortem. Muscle from cattle-fed RH, and supranutritional Zn had distinct differences in metabolic protein profiles that could directly impact metabolism and pH decline in postmortem muscle (Hochmuth et al, 2022). However, this relationship and differences in energy metabolism proteins have not been investigated in early postmortem muscle.

Investigations designed to define molecular factors associated with muscle growth and meat quality will inform efforts to improve and predict these important production features (Gagaoua et al., 2021; Huff-Lonergan and Lonergan, 2023; Kiyimba et al., 2023; Zhu et al., 2023). Defining the postmortem proteome, metabolome, and phosphoproteome can help elicit factors influencing the observed differences in pH decline and impacting tenderness. Therefore, this experiment aimed to identify the extent to which differences in the proteome, phosphorylation status of the proteome, and metabolome explain differences in pH decline and tenderness development. It was hypothesized that differences in the early postmortem metabolome and proteome (abundance and posttranslational modifications) of the longissimus thoracis (**LT**) muscle could influence pH decline and early postmortem tenderness development in beef from steers supplemented RH and supranutritional Zn.

## Materials and Methods

All procedures and protocols were approved by the Iowa State University Institutional Animal Care and Use Committee (11-17-8645-B).

### Sample collection

As previously described, 20 (*N* = 20) Angus crossbred steers were fed and harvested (approximately 15 to 16 mo of age), and LT rib sections were collected (Schulte et al., 2021). Briefly, steers were fed the same basal diet with an assignment to Zn and RH treatments. Zn treatments (SUPZN; basal Zn [36 mg Zn/kg DM analyzed] + 60 mg Zn/kg DM [ZnSO_4_] + 60 mg Zn/kg DM [as Zn–amino acid complex; Availa-Zn; Zinpro Corporation, Eden Prairie, MN]) were fed for the entire 89-d trial while RH treatments (300 mg per steer per d) were fed for the final 28 d before harvest. Final treatments consisted of a non-supplemented control (CON-NO; *n* = 5), supranutritional Zn-only supplemented (SUPZN-NO; *n* = 5), RH-only supplementation (CON-RAC; *n* = 5), and supranutritional Zn and RH supplementation (SUPZN-RAC; *n* = 5). Cattle were fed via GrowSafe bunks, monitoring each animal’s feed disappearance. At the conclusion of the feeding period, steers were harvested at the Iowa State University Meat Laboratory under USDA FSIS inspection. Postmortem LT muscle pH (1, 3, 6, and 24 h), WBSF values (1, 3, 7, and 14 d), and muscle samples (1 h and 1, 3, 7, and 14 d postmortem) were obtained and stored as described by Schulte et al. (2021). Postmortem pH and WBSF data were analyzed using the Mixed procedure of SAS version 9.4 (SAS Institute Inc., Cary, NC) as a factorial (2 × 2) with fixed effects of Zn and RH supplementation and their interaction. The harvest group was included as a fixed effect for analyses. Significance levels were set at *P ≤ *0.05 and trends 0.05 < *P ≤ *0.10.

### Whole muscle protein extraction

A frozen muscle sample containing only the LT (200 g) was homogenized and powdered in liquid nitrogen. Samples from each postmortem timepoint (0.5 g) were homogenized, and muscle proteins were solubilized using 10 mM sodium phosphate, pH 7.0, and 2% sodium dodecyl sulfate (SDS) (wt./vol) as described by Carlson et al. (2017b). Protein extractions were prepared in duplicate from all 1 h postmortem samples (*N* = 20).

### Sarcoplasmic protein extraction

Frozen meat containing only the LT (200 g) was homogenized and powdered in liquid nitrogen. Samples from each postmortem timepoint (3 g) were homogenized, and sarcoplasmic proteins were extracted (4 °C, 50 mM Tris-HCl, and 1 mM ethylenediaminetetraacetic acid [**EDTA**; pH 8.0]) as described by Carlson et al. (2017a). Samples for tandem mass tagging (**TMT**) analysis were diluted to 10 mg/mL using cold sarcoplasmic extraction buffer (4 °C, 50 mM Tris-HCl, and 1 mM EDTA [pH 8.0]) and were frozen until analysis. Protein extractions, in duplicate, were prepared from 1 h, 1, 3, 7, and 14 d postmortem samples.

### Troponin-T western blotting

Troponin-T western blots were conducted as described by Schulte et al. (2021). Differences in troponin-T degradation products were analyzed using the Mixed procedure of SAS (version 9.4, SAS Institute Inc.) as a factorial. Fixed effects of Zn, RH, and their interaction. Gel was used as a random effect in the model. Muscle protein extractions were utilized to complete Western blotting from 1 h, 1, 3, 7, and 14 d postmortem samples. Samples were run in at least duplicate until a coefficient of variation of less than 20% was achieved. Briefly, troponin-T was detected using monoclonal mouse anti-troponin-T primary antibody (T6277, JLT-12; Sigma-Aldrich, St. Louis, MO; 1:10,000) and goat anti-mouse HRP antibody (32430; Pierce, Thermo Scientific, Waltham, MA).

### Myosin heavy chain isoform analysis

Samples at 1 h postmortem were utilized to identify myosin heavy chain (**MHC**) isoforms described by Melody et al. (2004). Gels (0.75 mm thick) were loaded with 2 µg of protein per lane, run at 100 V for approximately 48 h, and silver stained (Silver Stain Plus Kit; Catalog #161-0449; Bio-Rad, Hercules, CA) for analysis. The density of type I and type IIa/IIx MHC isoforms were quantified using Alpha Ease FC software (version 3.03; Alpha Innotech, San Leandro, CA) and calculated as a percentage of the total MHC isoforms (MHC type I and type IIa + IIx) within each lane. Samples were run in at least duplicates with a coefficient of variance value of the bands of interest of 20% or less. Data were analyzed as a factorial (2 × 2) with a fixed effect of Zn and RH supplementation and a random effect of gel using the mixed procedure of SAS version 9.4 (v9.4, SAS Institute Inc.). Least squares means and standard errors were reported, and significance was set at *P ≤ *0.05 and trends 0.05 < *P ≤ *0.10. Muscle protein extractions were utilized to complete MHC isoforms analysis at 1 h postmortem. Samples were run in at least duplicate until a coefficient of variation of less than 20% was achieved.

### Muscle mineral analysis

Muscle samples from all samples (*N* = 20) at 7 d postmortem were homogenized and powdered in liquid nitrogen. Samples were prepared for analysis of trace minerals (Zn, copper, and iron) using 0.5 g of powdered muscle sample and analyzed via inductively coupled plasma optical emission spectroscopy (Optima 7000, Perkin-Elmer, Waltham, MA) as described by Pogge and Hansen (2013).

### TMT proteome analysis

Sarcoplasmic protein samples (all 1 h and 1 d postmortem; 200 µL, 10 mg/mL) were submitted to the Iowa State University Protein Facility (Ames, Iowa) for protein identification and quantification using TMT methods (Thompson et al., 2003; Zhai et al., 2020). Samples were run in duplicate. Samples were reduced using dithiothreitol, reduced cysteines were blocked using iodoacetamide, and digested overnight at 37 °C using trypsin/Lys-C (Promega, Madison, WI). Adding formic acid to each sample stopped digestion before drying in a Savant SpeedVac Plus SC110A with a Thermo Refrigerated Vapor Trap RVT4104 (Thermo Scientific). Samples were desalted using C18 MicroSpin Columns (Nest Group, Southborough, MA) and dried again in a Savant SpeedVac (Thermo Scientific). Samples were reconstituted in 1 M triethylammonium bicarbonate, and sample concentrations were determined using a Pierce Colorimetric kit (Fisher Scientific, Hampton, NH). Samples (25 µg of each) were labeled with TMT10plex reagents (Thermo Scientific). A pooled reference sample was prepared by pooling equal amounts of each sample, and this reference sample was labeled with TMT11 (131C) (Johnson et al., 2023a). Equal amounts of the labeled sample (2 µg) and the pooled reference (2 µg) were combined into a single tube and dried in a Savant SpeedVac. This combination of 10 TMT-labeled samples and a TMT-labeled reference sample was considered a single run (4 total runs in the current experiment). Samples were reconstituted in 5% acetonitrile/water/0.1% formic acid.

Peptides were separated by liquid chromatography (Thermo Scientific EASY nLC-1200 coupled to a Thermo Scientific Nanospray FlexIon source) through a pulled glass emitter 75 µm × 20 cm (Agilent, Santa Clara, CA). The emitter tip was packed with 5 µm SB-C18 packing material (Agilent), while the remainder of the column was packed with UChrom C18 3 µm material (nanoLCMS Solutions, Oroville, CA). A NanoSpray FlexIon source (Thermo Scientific) coupled to a Thermo Scientific Q Exactive Hybrid Quadrupole-Orbitrap Mass Spectrometer with a high-energy C-trap dissociation fragmentation cell was used to perform MS/MS. The resulting intact mass- and MS/MS fragmentation pattern was compared to MASCOT and Sequest HT theoretical fragmentation patterns to detect peptides that can identify proteins using Thermo Scientific’s Proteome Discoverer 2.4 software (Thermo Scientific). The MASCOT and Sequest HT search was run against Uniprot *Bos taurus*. Searches were performed with a static modification of carbamidomethyl (Cys) and TMT label (Lys and N-termini), along with dynamic modifications of oxidation (Met) and deamidation (Asn, Gln). Another search was performed with the additional dynamic modification of phosphorylation (Ser, Thr).

### Bioinformatics analysis

The signal intensity of each sample’s peptide was divided by the reference as the normalization factor. Peptides with greater than 50% missing values were removed from data analysis. The remaining data were normalized using the interquartile range estimation method (Vinutha et al., 2018). This process was repeated for each peptide in each treatment. MetaboAnalystR package v.3.0 (Pang et al., 2020) was used for data analysis. Upon finding data integrity to be satisfactory (positive values for the area), missing value estimations were imputed using the Singular Value Decomposition method (Pang et al., 2020). Non-informative values near constant throughout the experiment were detected using the interquartile range estimation method and deleted. Data were transformed based on generalized logarithm transformation to make individual features more comparable. Individual treatment comparisons were made at each postmortem timepoint (1 h and 1 d postmortem) by *t*-test with the *P*-value cutoff of 0.1. Log 2-fold change (**FC**) was performed to compare treatments. A FDR of 5% was used.

### Phosphoproteome analysis

The sarcoplasmic protein (10 mg/mL) fraction was utilized to identify phosphorylation modifications of proteins by using 2-dimensional gel electrophoresis. Samples were run in duplicate. Rehydration solution (DeStreak Catalog #17-6003-19; Cytiva, Marlborough, MA) was prepared using the manufacturer’s provided instructions by combining 2% immobilized pH gradient buffer (pH 4 to 7 [17-6000-86; Cytiva]) and 20 mM DTT. About 300 μg of protein (Immobiline DryStrip pH 4 to 7, 7 cm, Catalog #17-6001-10, Cytiva) were combined with rehydration solution and placed in individual strip holders (Catalog #80-6416-11; Cytiva). Strips were rehydrated for approximately 15 h in the dark at room temperature (22 °C). Strips were run for a total of 22,000 (4 to 7 pH) V-h on an Ettan IPGphor isoelectric focusing system (Catalog #11-0033-64; Cytiva). After running strips in the first dimension, strips were equilibrated in two 15-min washes. The first wash contained a mixture of equilibration buffer (50 mM Tris-HCl pH 8.8, 6 M urea, 30% glycerol, 2% SDS, and trace amounts of bromophenol blue) and 65 mM DTT. The second wash contained equilibration buffer and 135 mM iodoacetamide.

**Figure 1.**
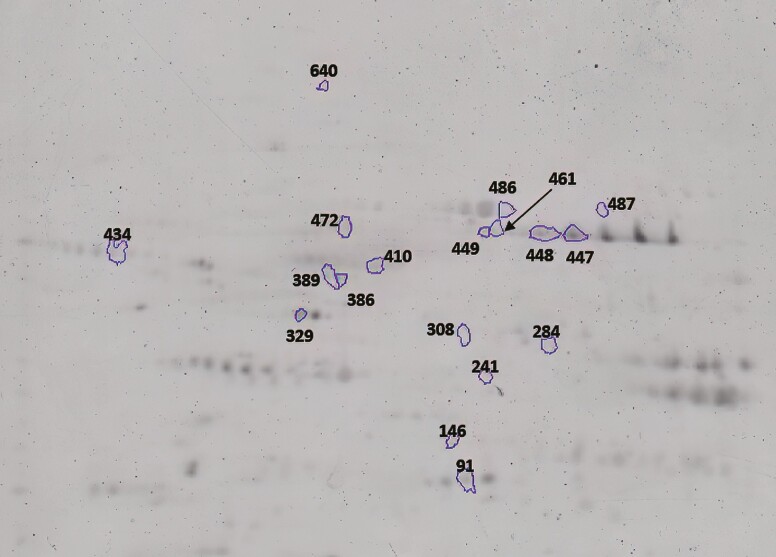
Representative 2-dimensional gel from the sarcoplasmic fraction of beef LT muscle at 1 h postmortem stained using Pro-Q Diamond stain. Immobilized pH gradient strips (24 cm, pH 4 to 7) were loaded with 100 µg of protein and strips were run on 12.5% SDS–polyacrylamide gel electrophoresis (PAGE)

Strips were then set in place on 12.5% preparative gels (10 cm wide × 10 cm tall × 1.5 mm thick; acrylamide: N,N’-bis-methylene acrylamide=100:1 [wt/wt], 0.1% [wt/vol] SDS, 0.05% [vol/vol] tetramethylene diamine (TEMED), 0.05% [wt/vol] ammonium persulfate (AMPER), 0.5 M Tris-HCl pH 8.8) using an overlay agarose with trace amounts of bromophenol blue. Gels were run at 120 V on SE 260 Hoefer Mighty Small II (Hoefer Inc., Holliston, MA) for approximately 1,000 V-h in running buffer. Gels were fixed, stained with phosphoprotein stain (Pro-Q Diamond, Fisher Scientific) and total protein stain (SYPRO Ruby; Fisher Scientific), and imaged on an Ettan DIGE Imager (GE Healthcare, Marlborough, MA) according to the manufacturer’s recommendations (Anderson et al., 2014). Figure 1 is a representative 7-cm, pH 4 to 7 phosphoprotein-stained gel. Using Melanie version 9.2.5 (Cytiva) software, gels were analyzed, and paired *t*-tests were conducted to identify phosphorylated protein abundance differences between treatments. The abundance of significantly different proteins between treatments was reported as FC (FC ≥ 1.25; *P* ≤ 0.10).

### Spot identification

Spots of interest between treatments were chosen for identification (FC ≥ 1.10; *P* ≤ 0.10). A pooled reference sample (500 µg) was used for protein spot identification. For spot identification, gels were run as described above to isolate spots of interest, stained with filtered Colloidal Coomassie Blue Stain (1.7% ammonium sulfate, 30% methanol, 3% phosphoric acid, and 0.1% Coomassie G-250), and destained using filtered double distilled water. Spots for identification were excised from gels, digested with trypsin at the Iowa State University Protein Facility, and identified as described by Schulte et al. (2020). Peptide fragments were compared to a known database program using Mascot (London, United Kingdom) and Sequest HT against *B. taurus* to identify proteins. Peptide fragments from identified proteins were analyzed for dynamic phosphorylation events at serine and threonine residues.

### Nontargeted metabolomics analysis

Total metabolite analysis was conducted at the W. M. Keck Metabolomics Research Laboratory (Ames, IA) as described by Jiye et al. (2005). Analysis was conducted using an Agilent Technologies Model 6890 gas chromatograph coupled with a model 5973 mass selective detector (GC-MS). All preparation steps were conducted on ice. Ribitol (10 µg; diluted in high-performance liquid chromatography grade water) and nonadecanoic fatty acid (10 µg; diluted in Hexane) were added to 50 mg of frozen tissue (1 h and 1 d). Methanol extraction solvent was added (8:2 MeOH: H_2_O) to each sample (0.9 mL). Samples were vortexed for 30 s, left on ice for 10 min, vortexed for another 30 s, and then left in an ice-cold sonicating water bath for 10 min. After sonication, samples were vortexed for 30 s and centrifuged (13,000 × *g*) for 7 min at room temperature (22 °C). The supernatant from each sample was then placed into GC-MS vials and frozen (−80 °C) until GC-MS derivatization.

Supernatants were thawed on ice and dried using a Savant SpeedVac SPD120 Vacuum Concentrator Kit (Thermo Scientific) overnight (~9 h). Samples were derivatized by adding 50 µl of freshly prepared methoxyamine hydrochloride (20 mg/mL in pyridine) and incubated (30 °C) for 90 min. Bis-trimethyl silyl trifluoroacetamide (70 µL) with 1% trimethylchlorosilane was added for silylation and incubated (60 °C) for 30 min.

Samples were fractionated (Agilent Technologies Model 6890 GC coupled to Model 5975 MS) and a hydrocarbon mix (C8-C40) was used as a retention index calibrator. Sample separation was completed on a GC-MS column (Agilent-HP5MSI; 30 m × 250 µM × 0.25 µM). The oven program used an initial temperature of 50 °C for 1 min, a 5 °C/min rate to 100 °C, and a 20 °C/min rate to 320 °C with a final hold for 5 min. Inlet and interface temperatures were controlled at 250 °C.

The detection mass range was set to 50–600 m/z. The Agilent ChemStation software controlled the GC-MS. The National Institutes of Standards and Technology mass spectral library (NIST, 2017) served as references for metabolites identified using the total ion mass spectrum. The MS peak area of each sample’s metabolites was divided by the reference (Ribitol) as the normalization factor. Metabolites that contained > 35% missing values were removed from the data set. The Half Minimum Method was used to replace missing values in the remaining metabolites (Wei et al., 2018). The statistical analysis module of MetaboAnalyst 5.0 (Chong et al. 2018; Xia Lab, McGill, CA) was used to analyze metabolites. Data integrity was checked utilizing MetaboAnalyst 5.0 (Chong et al. 2018; Xia Lab). Data were transformed based on generalized logarithm transformation to make individual features more comparable. Individual treatment comparisons were made within each postmortem timepoint (1 h and 1 d postmortem) by t-test with the *P*-value cutoff of 0.1. Log 2-FC analysis was performed to compare the FC between treatments.

## Results and Discussion

### Phenotypic summary

Feeding supplemental Zn and RH to beef steers affected the postmortem LT muscle pH decline and WBSF values at 1 d postmortem (Schulte et al., 2021). Zn supplementation tended to result in lower (*P* = 0.06) pH values at 6 h postmortem and lower (*P* = 0.06) WBSF values at 1 d postmortem (Table 1), whereas RH supplementation resulted in greater (*P* = 0.04) pH values at 6 h postmortem and greater (*P* < 0.01) WBSF values at 1 d postmortem (Table 1). Greater WBSF values in steaks from steers fed RH were attributed to less postmortem protein degradation of whole muscle troponin-T at 1 d postmortem (Table 1). Tough pork chops showed more intact desmin in the myofibrillar fraction than tender pork chops (Johnson et al. 2023b), suggesting less protein degradation with less tender meat. As postmortem metabolism and pH decline were influenced by ZN and RH supplementation (Schulte et al., 2021), further investigation into the proteome and metabolome of this muscle is necessary to more fully understand what metabolic pathways and proteome differences exist early postmortem to explain the observed pH decline and WBSF differences.

**Table 1.** Effect of supranutritional zinc (Zn) and RH supplementation on pH decline, WBSF, troponin-T degradation, and MHC isoform data of beef LT muscle

	CON^1^		SUPZN^1^		*P*-value		
Item	NO^2^	RAC^2^	NO^2^	RAC^2^	ZNTRT	RACTRT	ZNTRT × RACTRT
pH decline							
1 h	6.48 (0.06)	6.60 (0.06)	6.48 (0.06)	6.55 (0.06)	0.92	0.13	0.58
3 h	6.00 (0.07)	6.17 (0.08)	5.92 (0.08)	6.02 (0.07)	0.16	0.11	0.65
6 h	5.68 (0.08)	5.86 (0.08)	5.49 (0.08)	5.70 (0.08)	0.06	0.04	0.90
24 h	5.48 (0.07)	5.46 (0.07)	5.48 (0.07)	5.47 (0.07)	0.89	0.75	0.93
WBSF^3^							
1 d	6.7 (0.4)	7.6 (0.4)	5.4 (0.4)	7.4 (0.4)	0.06	<0.01	0.16
3 d	4.7 (0.3)	5.4 (0.3)	4.6 (0.3)	5.2 (0.3)	0.73	0.11	0.88
7 d	3.3 (0.4)	4.0 (0.4)	4.2 (0.4)	4.0 (0.4)	0.11	0.43	0.17
14 d	3.4 (0.3)	3.4 (0.3)	3.6 (0.3)	3.8 (0.3)	0.42	0.74	0.84
Troponin-T degradation^4^							
1 d	1.29 (0.24)	0.68 (0.24)	1.09 (0.23)	0.65 (0.26)	0.64	0.04	0.74
3 d	3.44 (0.53)	2.59 (0.49)	2.38 (0.53)	1.60 (0.48)	0.05	0.11	0.95
7 d	4.93 (0.61)	4.56 (0.55)	3.71 (0.59)	4.55 (0.54)	0.16	0.59	0.18
14 d	7.55 (0.89)	6.20 (0.76)	4.78 (0.79)	4.31 (0.79)	<0.01	0.27	0.59
MHC isoform, %^5^							
Type I	60.96 (1.78)	58.93 (1.78)	62.49 (1.91)	59.48 (1.73)	0.57	0.17	0.79
Type IIa + IIx	39.04 (1.78)	41.07 (1.78)	37.51 (1.91)	40.52 (1.73)	0.57	0.17	0.79
Trace mineral analysis, mg/kg DM							
Copper	1.2 (0.6)	1.2 (0.5)	2.3 (0.5)	1.1 (0.6)	0.38	0.27	0.33
Iron	52 (4)	50 (3)	54 (3)	55 (4)	0.35	0.90	0.66
Zinc	134 (8)	132 (7)	132 (7)	128 (8)	0.73	0.72	0.92

^1^CON = no supplemental Zn (analyzed 36 mg Zn/kg DM); SUPZN = CON + 60 mg Zn/kg DM from ZnSO4 + 60 mg Zn/kg DM from Zn–amino acid complex (Availa-Zn; Zinpro Corporation). Fed for the entire 89 d trial.

^2^NO = no supplemental RH; RH = 300 mg RH per head per d (Actogain45; Zoetis, Parsippany, NJ) starting 28 d prior to harvest.

^3^WBSF values (kg) were averaged across 2 adjacent steaks.

^4^Ratio of the densitometry units of the degraded 30-kDa band of the sample compared with the 30-kDa band of the reference sample.

^5^Proportion of MHC detected as type I or IIa + IIx on SDS-polyacrylamide gel electrophoresis (PAGE) migration. Protein samples were prepared from LT muscle removed from carcass at 1 h postmortem.

Means and standard errors of the mean are reported.

CON-NO = No Zn (36 mg/kg DM basis) or RH supplementation. CON-RAC = No Zn supplementation, only RH supplementation (300 mg/steer/d for 28 d). SUPZN-NO = Zn supplementation (60 mg Zn/kg DM as ZnSO4 + 60 mg Zn/kg DM as Zn–amino acid), no RH supplementation. SUPZN-RAC = Zn and RH supplementation.

### MHC isoform analysis

Fiber type can influence muscle metabolism (Schiaffino and Reggiani, 2011). MHC isoform analysis (type I and type IIa + IIx) was utilized to identify differences in MHC isoforms which could identify fiber type differences. No differences in MHC isoforms between treatments or their interaction were detected in the current study (Table 1; *P* > 0.17). The impacts of supplementation of RH (Gonzalez et al., 2007, 2009; Ebarb et al., 2017) and Zn (Hergenreder et al., 2016; Wellmann et al., 2020) on MHC isoforms are inconsistent. The differences in RH and Zn supplementation effects on MHC isoforms can be due to the analysis method, animal age, muscle, and absorption of supplementations between studies. This lack of difference further solidifies the need to identify proteome and metabolome differences that influence postmortem metabolism and tenderness development in treatments.

### Proteome and metabolome analysis: SUPZN-NO

The steaks from the SUPZN-NO treatment group had the most rapid pH decline and the lowest WBSF values at day 1 (2.2 kg less than steaks in the CON-RAC treatment group; Table 1). Several interesting metabolites and proteins were uniquely different in the SUPZN-NO treatment compared to the other 3 treatments. In the metabolomic analysis, a greater abundance of sorbitol was identified in SUPZN-NO at 1 d postmortem than in any other treatment (Table 2; *P* ≤ 0.03). In proteomic analysis, a greater abundance of zeta-crystallin (Table 3; *P* ≤ 0.04) and less abundance of myosin regulatory light chain (MYL) 2 was identified in SUPZN-NO compared to all other treatments at 1 h postmortem (Table 4; *P* ≤ 0.09).

**Table 2.** Effect of supranutritional zinc (Zn) and RH supplementation on the beef LT muscle metabolome at 1 d postmortem

Pathway	Metabolite	Comparison^1^^,^^2^	Log 2-FC^3^	*P*-value
Glycolysis	Glucose	CON-RAC/CON-NO	0.67	0.05
Glycolysis	Glucose-6-phosphate	CON-RAC/CON-NO	0.53	0.08
Polyol pathway	Fructose	CON-RAC/CON-NO	1.24	0.05
Amino acid	Glycine	CON-RAC/CON-NO	0.95	0.08
Other	Hydroxybutyric acid	CON-RAC/CON-NO	0.78	0.09
Polyol pathway	Sorbitol	SUPZN-NO/CON-NO	1.40	0.02
Polyol pathway	Fructose	SUPZN-NO/CON-NO	0.66	0.05
Amino acid	l-5-Oxoproline	SUPZN-NO/CON-NO	0.86	0.06
Glycolysis	Per-triethylsilydehydro derivative of glucose	SUPZN-NO/CON-NO	1.06	0.06
Glycolysis	Fructose-6-phosphate	SUPZN-NO/CON-NO	1.09	0.08
Glycolysis	Glucose	SUPZN-NO/CON-NO	1.17	0.09
Fatty acid	Oleic acid	SUPZN-NO/CON-NO	−0.80	0.09
Other	Hydroxybutyric acid	SUPZN-NO/CON-NO	1.03	0.001
Other	RI 1930.8 RT 17.3778 328D	SUPZN-NO/CON-NO	1.92	0.04
Other	RI 1863.8 RT 17.0314 332D	SUPZN-NO/CON-NO	1.06	0.06
Other	Erythronic acid	SUPZN-NO/CON-NO	1.31	0.09
Fatty acid	Oleic acid	SUPZN-RAC/CON-NO	−0.85	0.01
Amino acid	l-5-Oxoproline	SUPZN-RAC/CON-NO	1.01	0.03
Glycolysis	Per-triethylsilydehydro derivative of glucose	SUPZN-RAC/CON-NO	0.94	0.08
Polyol pathway	Sorbitol	SUPZN-NO/CON-RAC	2.07	0.01
Glycolysis	Glucose-6-phosphate	SUPZN-NO/CON-RAC	−0.53	0.05
Glycolysis	Lactate	SUPZN-NO/CON-RAC	0.50	0.05
Glycolysis	Fructose-6-phosphate	SUPZN-NO/CON-RAC	1.21	0.08
Glycolysis	Glucose	SUPZN-NO/CON-RAC	−0.46	0.08
Amino acid	Glycine	SUPZN-NO/CON-RAC	−0.84	0.09
Fatty acid	Linoleic acid	SUPZN-NO/CON-RAC	0.82	0.10
Other	RI 1863.8 RT 17.0314 332D	SUPZN-NO/CON-RAC	1.89	0.005
Other	Glycerol	SUPZN-NO/CON-RAC	0.63	0.01
Other	Phosphoric acid	SUPZN-NO/CON-RAC	−0.61	0.02
Other	Fructofuranose	SUPZN-NO/CON-RAC	1.74	0.02
Other	RI 1930.8 RT 17.3778 328D	SUPZN-NO/CON-RAC	2.31	0.02
Other	Myo-inositol	SUPZN-NO/CON-RAC	−1.01	0.03
Other	Propylene glycol	SUPZN-NO/CON-RAC	1.25	0.08
Other	Diethylcarbamate	SUPZN-NO/CON-RAC	0.79	0.1
Amino acid	RI 1439.4 RT 14.3244 328D	SUPZN-RAC/CON-RAC	−0.89	0.04
Fatty acid	Linoleic acid	SUPZN-RAC/CON-RAC	0.79	0.08
Polyol pathway	Sorbitol	SUPZN-RAC/SUPZN-NO	−1.04	0.03
Other	RI 1863.8 RT 17.0314 332D	SUPZN-RAC/SUPZN-NO	−1.32	0.02
Other – phosphate	Phosphoric acid	SUPZN-RAC/SUPZN-NO	0.65	0.03
Other	Glycerate	SUPZN-RAC/SUPZN-NO	0.48	0.04
Other	Hydroxybutyric acid	SUPZN-RAC/SUPZN-NO	−0.56	0.04
Other	Boric acid	SUPZN-RAC/SUPZN-NO	−1.38	0.09

^1^CON = no supplemental Zn (analyzed 36 mg Zn/kg DM); SUPZN = CON + 60 mg Zn/kg DM from ZnSO4 + 60 mg Zn/kg DM from Zn–amino acid complex (Availa-Zn; Zinpro Corporation). Fed for the entire 89 d trial.

^2^NO = no supplemental RH; RH = 300 mg RH per head per d (Actogain45; Zoetis, Parsippany, NJ) starting 28 d prior to harvest.

^3^Log 2-FC indicates metabolite abundance differences between treatments. Positive values are more abundant in the treatment comparison identified first in the comparison column. Negative values are less abundant in the treatment comparison identified first in the comparison column.

CON-NO = No Zn (36 mg/kg DM basis) or RH supplementation. CON-RAC = No Zn supplementation, only RH supplementation (300 mg/steer/d for 28 d). SUPZN-NO = Zn supplementation (60 mg Zn/kg DM as ZnSO4 + 60 mg Zn/kg DM as Zn–amino acid), no RH supplementation. SUPZN-RAC = Zn and RH supplementation.

**Table 3.** Other proteins differing between nutritional treatments at 1 h and 1 d postmortem

Protein	Accession number	Gene	Coverage, %	Unique peptides	Log 2-FC^1^	*P*-value	Location	Comparison
1 h								
Myoglobin	A0A1K0FUF3	GLNG	99	27	−0.17	0.09	Sarcoplasm	CON-RAC/CON-NO
Zeta-crystallin	O97764	CRYZ	53	12	0.28	0.04	Sarcoplasm	SUPZN-NO/CON-NO
Zeta-crystallin	O97764	CRYZ	53	12	0.34	0.02	Sarcoplasm	SUPZN-NO/CON-RAC
Zeta-crystallin	O97764	CRYZ	53	12	−0.45	<0.01	Sarcoplasm	SUPZN-RAC/SUPZN-NO
1 d								
Myoglobin	A0A1K0FUF3	GLNG	99	27	−0.26	0.09	Sarcoplasm	CON-RAC/CON-NO

^1^Log 2-FC indicates protein abundance differences between treatments. Positive values are more abundant in the treatment comparison identified first in the comparison column. Negative values are less abundant in the treatment comparison identified first in the comparison column.

CON = no supplemental Zn (analyzed 36 mg Zn/kg DM); SUPZN = CON + 60 mg Zn/kg DM from ZnSO4 + 60 mg Zn/kg DM from Zn–amino acid complex (Availa-Zn; Zinpro Corporation). Fed for the entire 89 d trial.

NO = no supplemental RH; RH = 300 mg RH per head per d (Actogain45; Zoetis, Parsippany, NJ) starting 28 d prior to harvest.

CON-NO = No Zn (36 mg/kg DM basis) or RH supplementation. CON-RAC = No Zn supplementation, only RH supplementation (300 mg/steer/d for 28 d). SUPZN-NO = Zn supplementation (60 mg Zn/kg DM as ZnSO4 + 60 mg Zn/kg DM as Zn–amino acid), no RH supplementation. SUPZN-RAC = Zn and RH supplementation.

**Table 4.** Structural proteins differing between nutritional treatments at 1 h postmortem

Protein	Accession number	Gene	Coverage, %	Unique peptides	Log 2-FC^1^	*P*-value	Location	Comparison
Actin, alpha-1 skeletal muscle	A4IFM8	ACTA1	59	8	0.30	0.01	Sarcomere	CON-RAC/CON-NO
Troponin-T, slow skeletal muscle	A0A3Q1MFN5	TNNT1	19	3	0.30	0.04	Sarcomere	CON-RAC/CON-NO
Tropomyosin alpha-1 chain	Q5KR49	TPM1	59	10	0.41	0.08	Sarcomere	CON-RAC/CON-NO
Myosin-7	F1MM07	MYH7	44	50	0.55	0.10	Sarcomere	CON-RAC/CON-NO
Myosin regulatory light chain 2	A0A3Q1N6P5	MYL2	52	6	−0.25	0.09	Sarcomere	SUPZN-NO/CON-NO
Myosin light polypeptide 6	P60661	MYL6	25	2	−0.30	0.03	Sarcomere	SUPZN-NO/CON-NO
Myosin light chain kinase 2	A4IFM7	MYLK2	49	18	0.22	0.02	Sarcomere	SUPZN-RAC/CON-NO
Myosin-7	F1MM07	MYH7	44	50	0.93	0.03	Sarcomere	SUPZN-RAC/CON-NO
Myosin heavy chain 1	Q9BE40	MYH1	48	10	0.74	0.05	Sarcomere	SUPZN-RAC/CON-NO
Myosin heavy chain 4	E1BP87	MYH4	35	2	1.58	0.07	Sarcomere	SUPZN-RAC/CON-NO
Troponin-T, slow skeletal muscle	A0A3Q1MFN5	TNNT1	19	3	0.36	0.09	Sarcomere	SUPZN-RAC/CON-NO
Myosin regulatory light chain 2	A0A3Q1N6P5	MYL2	52	6	−0.81	0.07	Sarcomere	SUPZN-NO/CON-RAC
Actin	P60712	ACTB	49	5	0.14	0.08	Sarcomere	SUPZN-RAC/CON-RAC
Myosin regulatory light chain 2	A0A3Q1N6P5	MYL2	52	6	0.54	0.04	Sarcomere	SUPZN-RAC/SUPZN-NO
Troponin C, slow skeletal	P63315	TNNC1	27	4	0.22	0.04	Sarcomere	SUPZN-RAC/SUPZN-NO
Tropomyosin alpha-3 chain	Q5KR47	TPM3	49	8	0.36	0.05	Sarcomere	SUPZN-RAC/SUPZN-NO
Myosin light polypeptide 6	P60661	MYL6	25	2	0.28	0.06	Sarcomere	SUPZN-RAC/SUPZN-NO
Actin, cytoplasmic 1	P60712	ACTB	49	5	0.16	0.06	Sarcomere	SUPZN-RAC/SUPZN-NO
Desmin	O62654	DES	43	14	0.35	0.09	Sarcomere, nucleus, sarcoplasm	SUPZN-RAC/SUPZN-NO

^1^Log 2-FC indicates protein abundance differences between treatments. Positive values are more abundant in the treatment comparison identified first in the comparison column. Negative values are less abundant in the treatment comparison identified first in the comparison column.

CON = no supplemental Zn (analyzed 36 mg Zn/kg DM); SUPZN = CON + 60 mg Zn/kg DM from ZnSO4 + 60 mg Zn/kg DM from Zn–amino acid complex (Availa-Zn; Zinpro Corporation). Fed for the entire 89 d trial.

NO = no supplemental RH; RH = 300 mg RH per head per d (Actogain45; Zoetis, Parsippany, NJ) starting 28 d prior to harvest.

CON-NO = No Zn (36 mg/kg DM basis) or RH supplementation. CON-RAC = No Zn supplementation, only RH supplementation (300 mg/steer/d for 28 d). SUPZN-NO = Zn supplementation (60 mg Zn/kg DM as ZnSO4 + 60 mg Zn/kg DM as Zn–amino acid), no RH supplementation. SUPZN-RAC = Zn and RH supplementation.

A greater abundance of sorbitol indicates alternative energy metabolism pathways utilized in muscle from cattle supplemented SUPZN-NO (Table 2; *P* ≤ 0.03). The polyol pathway produces sorbitol. This pathway decreases ATP production (Cotter et al., 1993) and is primarily utilized when excess glucose exists in the system and must be cleared in the muscle. The first step utilizes aldose reductase, with the consumption of nicotinamide adenine dinucleotide phosphate (NADPH), to convert glucose to sorbitol (Gallagher et al., 2016). The second step utilizes sorbitol dehydrogenase and nicotinamide adenine dinucleotide as electron acceptors to produce fructose from sorbitol (Brownlee, 2005). Interestingly, fructose content was also greater in SUPZN-NO than CON-NO at 1 d postmortem (Table 2; *P = *0.05). Lastly, greater glucose was identified in SUPZN-NO than CON-NO at 1 d postmortem (Table 2; *P = *0.09). These identified increased glucose conditions in the SUPZN-NO treatment and potential utilization of the polyol pathway, which may be influencing the differences in pH decline observed between treatments.

Free glucose is transported into muscle by glucose transporter 4 (GLUT4; Chadt and Al-Hasani, 2020). To transport free glucose into the muscle cell, GLUT4 must translocate to the plasma membrane. Translocation of GLUT4 is initiated by insulin- and contraction-responsive mechanisms (Chadt and Al-Hasani, 2020), which can be mimicked by the function of Zn (Y. Wu et al., 2016). In living muscle, supplementation of supranutritional Zn compared to no additional Zn supplementation has been hypothesized to decrease muscle fatigue by reducing the need to clear lactate buildup in muscle and glucose production during and after transit (Heiderscheit and Hansen, 2022). Post-transit, Zn treatments had less muscle glucose and GLUT4 compared to the control treatment that was not supplemented additional Zn. Lesser muscle glucose in Zn treatments could indicate a lesser extent of glycogen depletion and GLUT4 expression in living muscle but the impacts of these feeding strategies in postmortem muscle have not been defined.

The impacts of Zn supplementation on postmortem muscle were investigated in the current study where greater glucose content in SUPZN-NO compared to CON-NO could be a function of Zn to bring free glucose into the muscle or glucose produced from the deterioration of glucose-6-phosphate into glucose through glycogenolysis. Although total Zn content in the muscle was not greater in SUPZN-NO in the current study (Table 1; *P = *0.73), localization of Zn in membranes or organelles could assist with understanding the role of Zn in muscle (antemortem and postmortem) to influence postmortem pH decline. Regulation of skeletal muscle glucose is complex (Villar-Palasi and Guinovart, 1997) thus further investigation into this potential role is needed.

The SUPZN-NO treatment had a less adenosine monophosphate deaminase 1 (AMPD1) than CON-NO and SUPZN-RAC at 1 d postmortem (Table 5; *P = *0.03 and *P* = 0.04, respectively). Although only significant in 2 out of the 3 contrasts, in the contrast of SUPZN-NO and CON-RAC, SUPZN-NO had a lower abundance of AMPD1 following the same trends as the other comparisons. AMPD1 is a Zn-containing metalloenzyme (Stankiewicz et al., 1980) responsible for the deamination of adenosine monophosphate (AMP) to inosine monophosphate (IMP) and ammonia (Lushchak, 1996). As an essential part of maintaining homeostasis of the adenine nucleotide pool, AMPD1 plays a key role in postmortem energy production and the extent to which nucleotides can be recycled for energy production as deamination can limit muscle pH decline (England et al., 2015). Glycogenolysis and glycolysis are stimulated by AMP. When AMPD1 is inhibited and AMP accumulates, pH declines further, more lactic acid is accumulated, and glycogen catabolism occurs at a greater rate (England et al., 2015). This may provide an explanation for the observation of Zhu et al (2023) who demonstrated a positive correlation between AMPD1 abundance and beef sensory flavor. The activity of AMPD1 is enhanced when the enzyme is phosphorylated (Rush et al., 1998; Tullson et al., 1998). Less phosphorylated AMPD1 at 1 d postmortem in the sarcoplasmic fraction was found in the Zn-only treatment (Table 5; *P* ≤ 0.04) predicting more accumulation of AMP and more rapid pH decline. AMPD1 contains a histidine-rich glycoprotein which can form a cluster with one Zn molecule and 3 histidine’s (Mangani et al., 2003, 2007). Through Zn-affinity chromatography, separation of this histidine-rich glycoprotein reduced the solubility of AMPD (Ranieri-Raggi et al., 2003). This Zn-binding site is located in the catalytic site of AMPD and thus could influence the structure and activity of this enzyme. Further investigation into the influence of Zn supplementation on AMPD1 abundance and activity is necessary to understand how AMPD1 may impact energy metabolism, pH decline, and tenderness development.

**Table 5.** Energy metabolism proteins differing between nutritional treatments at 1 h and 1 d postmortem

Protein	Accession number	Gene	Coverage, %	Unique peptides	Log 2-FC^1^	*P*-value	Location	Comparison
1 h								
Glyceraldehyde-3-phosphate dehydrogenase	Q2KJE5	GAPDHS	13	2	0.17	0.08	Sarcoplasm	CON-RAC/CON-NO
Glyceraldehyde-3-phosphate dehydrogenase	P10096	GAPDH	80	15	−0.19	0.04	Sarcomere, nucleus, cytosol	SUPZN-RAC/CON-RAC
Glyceraldehyde-3-phosphate dehydrogenase	Q2KJE5	GAPDHS	13	2	0.20	0.07	Sarcoplasm	SUPZN-RAC/SUPZN-NO
ATP synthase subunit delta	P05630	ATP5F1D	14	2	−2.30	0.07	Mitochondria	SUPZN-NO/CON-RAC
1 d								
Glyceraldehyde-3-phosphate dehydrogenase	P10096	GAPDH	80	15	0.21	0.08	Sarcoplasm, sarcomere, nucleus	CON-RAC/CON-NO
Glyceraldehyde-3-phosphate dehydrogenase	P10096	GAPDH	80	15	−0.15	0.10	Cytosol, nucleus, sarcomere	SUPZN-RAC/CON-RAC
AMP deaminase	F1MLX6	AMPD1	7	5	−0.51	0.03	Cytosol	SUPZN-NO/CON-NO
AMP deaminase	F1MLX6	AMPD1	7	5	0.29	0.04	Cytosol	SUPZN-RAC/SUPZN-NO
ATP synthase subunit beta	A0A452DII8	ATP5F1B	37	14	1.05	0.04	Mitochondria	SUPZN-NO/CON-RAC
ATP synthase subunit b	A0A3Q1MP66	N/A	3	1	1.90	0.07	Mitochondria	SUPZN-NO/CON-RAC
Malate dehydrogenase	Q32LG3	MDH2	64	17	0.22	0.07	Mitochondria	SUPZN-NO/CON-NO
Malate dehydrogenase	Q32LG3	MDH2	64	17	−0.17	0.06	Mitochondrial	SUPZN-RAC/SUPZN-NO

^1^Log 2-FC indicates protein abundance differences between treatments. Positive values are more abundant in the treatment comparison identified first in the comparison column. Negative values are less abundant in the treatment comparison identified first in the comparison column.

CON = no supplemental Zn (analyzed 36 mg Zn/kg DM); SUPZN = CON + 60 mg Zn/kg DM from ZnSO4 + 60 mg Zn/kg DM from Zn–amino acid complex (Availa-Zn; Zinpro Corporation). Fed for the entire 89 d trial.

NO = no supplemental RH; RH = 300 mg RH per head per d (Actogain45; Zoetis, Parsippany, NJ) starting 28 d prior to harvest.

CON-NO = No Zn (36 mg/kg DM basis) or RH supplementation. CON-RAC = No Zn supplementation, only RH supplementation (300 mg/steer/d for 28 d). SUPZN-NO = Zn supplementation (60 mg Zn/kg DM as ZnSO4 + 60 mg Zn/kg DM as Zn–amino acid), no RH supplementation. SUPZN-RAC = Zn and RH supplementation.

Phosphoglucomutase-1 (PGM1) is a key glycolytic enzyme responsible for the interconversion of glucose-1-phosphate to glucose-6-phosphate using the intermediate compound, glucose-1,6-bisphosphate. Phosphorylation of PGM1 at threonine 466 has been demonstrated to increase the enzyme activity of PGM1 (Gururaj et al., 2004). PGM1 is a consistent biomarker of beef tenderness due to abundance and phosphorylation differences (Anderson et al., 2012, 2014; Picard et al., 2018; Silva et al., 2019; Picard and Gagaoua, 2020a, b; Gagaoua et al., 2021). PGM1 total abundance is a potential biomarker of tenderness and has identified positive and negative associations with beef tenderness depending on muscle and sex (Picard and Gagaoua, 2020a, b; Gagaoua et al., 2021). Two-dimensional difference in gel electrophoresis was utilized to identify proteome differences in beef longissimus dorsi muscle classified based on 14 d postmortem tenderness values (Anderson et al., 2012). Five spots of PGM1 were significantly different (Anderson et al., 2012), with 4 being greater in abundance in the tender classification and one (most alkaline) being more abundant in the tough classification, In a follow-up study, Anderson et al. (2014) identified in beef longissimus dorsi muscle that the more tender classification had a lesser total and phosphorylated abundance of the least phosphorylated (most alkaline) isoform of PGM1.

The current study identified a greater abundance of PGM1 in all 3 spots (Spot 447 [*P = *0.06], 448 [*P = *0.03], and 472 [*P = *0.03]) in the CON-RAC and SUPZN-NO treatments compared to CON-NO in the 2-dimensional gel phosphoproteome analysis (Table 6; Fig. 1). Additionally, a greater abundance of PGM1 (spot 448) was identified in CON-RAC than SUPZN-NO. Lastly, CON-RAC and SUPZN-NO had a greater abundance of PGM1 (spot 448) than SUPZN-RAC.

**Table 6.** Phosphoproteome comparison at 1 h postmortem of muscle from cattle supplemented supranutritional zinc (Zn) and RH

Spot #	Protein	Accession number	Gene	Coverage, %	Unique peptides	Fold change						ZNTRT	RACTRT	ZNTRT × RACTRT	Location
						CON-RAC/CON-NO	SUPZN-NO/CON-NO	SUPZN-RAC/CON-NO	SUPZN-NO/CON-RAC	SUPZN-RAC/CON-RAC	SUPZN-RAC/SUPZN-NO				
Energy production															
91	Malate Dehydrogenase	Q3T145	MDH1	45	15	1.58	0.94	0.91	0.59	0.57	0.97	0.02	0.08	0.05	Cytoplasm
241	Succinate—CoA ligase subunit beta	F1MGC0	SUCLA2	43	22	1.06	0.94	0.66	0.88	0.63	0.71	0.04	0.33	0.13	Mitochondria
308	4-trimethylaminobutyraldehyde dehydrogenase	Q2KJH9	ALDH9A1	30	14	1.32	0.87	0.74	0.66	0.56	0.85	0.04	0.56	0.18	Cytosol
329	ATP synthase subunit beta	A0A4W2EL77	ATP5F1B	48	19	1.58	1.17	0.88	0.74	0.55	0.75	0.10	0.36	0.01	Mitochondria, membranes
447	Phosphoglucomutase-1	A0A3Q1LRD1	PGM1	62	1	1.39	1.46	0.78	1.05	0.56	0.54	0.78	0.60	0.06	Sarcoplasm
448	Phosphoglucomutase-1	A0A4W2F139	PGM1	64	1	1.41	1.78	0.94	1.27	0.67	0.53	0.56	0.42	0.03	Sarcoplasm
472	Phosphoglucomutase-1	A0A4W2F139	PGM1	27	12	1.68	1.28	0.86	0.76	0.51	0.67	0.26	0.58	0.03	Sarcoplasm
146	Aldose reductase	A0A4W2GXB1	LOC113891189	34	9	1.04	0.96	0.64	0.93	0.61	0.66	0.01	0.06	0.02	Cytosol
Structural															
386	Actin	P68138	ACTA1	41	5	1.68	1.44	0.92	0.85	0.55	0.64	0.51	0.74	0.03	Sarcomere
Phosphorylation															
410	T1-TrpRS	A0A4W2HQN8	WARS1	19	7	1.09	1.43	1.76	1.31	1.61	1.23	0.04	0.41	0.63	Nucleus, cytosol
Lipid homeostasis															
434	Alpha-2-HS-glycoprotein	P12763	AHSG	22	1	0.88	0.69	0.70	0.78	0.80	1.03	0.01	0.55	0.42	Secreted
Apoptosis															
449	Heat shock 70 kDa protein 1A	K9ZRL4	HSPA1A	45	1	0.73	1.07	0.66	1.46	0.90	0.62	0.99	0.01	0.54	Nucleoplasm, vesicles, cytosol
461	Heat shock 70 kDa protein 1A	K9ZRL4	HSPA1A	42	14	0.81	1.14	0.67	1.40	0.82	0.59	0.98	0.01	0.26	Nucleoplasm, vesicles, cytosol
486	Albumin	P02769	ALB	70	45	1.08	0.78	0.61	0.72	0.57	0.79	0.04	0.79	0.44	Secreted
640	Heat shock 70 kDa protein 4	A0A4W2FG09	HSPA4	34	23	1.17	0.71	1.43	0.60	1.21	2.02	0.87	0.00	0.04	Sarcoplasm
Other															
284	Rab GDP dissociation inhibitor	A0A4W2CLS3	GDI2	49	17	1.51	1.30	1.12	0.86	0.74	0.86	0.70	0.19	0.01	Sarcoplasm
389	Serpin family A member 1	A0A4W2DYT6	SERPINA1	30	13	1.55	1.27	1.02	0.82	0.65	0.80	0.39	0.34	0.02	Endoplasmic reticulum
487	Beta-1 metal-binding globulin	A0A4W2DEN7	TF	44	1	0.67	0.59	1.06	0.87	1.58	1.80	0.94	0.70	0.04	Secreted

CON = no supplemental Zn (analyzed 36 mg Zn/kg DM); SUPZN = CON + 60 mg Zn/kg DM from ZnSO4 + 60 mg Zn/kg DM from Zn–amino acid complex (Availa-Zn; Zinpro Corporation). Fed for the entire 89 d trial.

NO = no supplemental RH; RH = 300 mg RH per head per d (Actogain45; Zoetis, Parsippany, NJ) starting 28 d prior to harvest.

The dynamic modification of phosphorylation (Ser, Thr) were analyzed for each protein.

CON-NO = No Zn (36 mg/kg DM basis) or RH supplementation. CON-RAC = No Zn supplementation, only RH supplementation (300 mg/steer/d for 28 d). SUPZN-NO = Zn supplementation (60 mg Zn/kg DM as ZnSO4 + 60 mg Zn/kg DM as Zn–amino acid), no RH supplementation. SUPZN-RAC = Zn and RH supplementation.

Within spot 447, phosphorylation sites were identified at serine 117, 134, and 505 in the polypeptide. Within spot 448, the same 3 phosphorylation events were found on each serine with an additional phosphorylation event at threonine 19. These differences in phosphoproteome analysis of PGM1 spots are primarily in the individual feeding treatments (CON-RAC and SUPZN-NO) which were the most different in pH decline (SUPZN-NO = 5.49 at 6 h postmortem; CON-RAC = 5.86 at 6 h postmortem). This demonstrates that specific isoforms of PGM1 coincide with treatments with a slower pH decline. Specifically, samples in the CON-RAC treatment had a greater abundance of spot 448 (the most acidic spot in the PGM1 train of spots and phosphorylation at serine 117, 134, 505, and threonine 19). As PGM1 is a strong candidate protein for tenderness identification, further investigation in phosphorylation and other posttranslational modifications is needed. As phosphorylation can enhance the activity of PGM1, this could enhance postmortem metabolism and thus result in differences in pH decline and thus earlier arrest of rigor in muscle and differences in tenderness development as seen in the current study.

Through the first step of the polyol pathway, NADPH is consumed to convert glucose to sorbitol with the activity of aldose reductase. This step affects the oxidative status of the muscle by removing NADPH (an antioxidant), which is utilized to generate glutathione and thus prevent reactive oxygen species scavenging (Brownlee, 2005; Sánchez et al., 2009). As sorbitol production can influence the oxidative status of muscle, this influence was demonstrated in proteomic analysis. At 1 d postmortem, SUPZN-NO had greater peroxiredoxin-1 (*P = *0.03), peroxiredoxin-3 (*P = *0.01), and glutathione S-transferase P (*P = *0.06) than CON-NO (Table 7), demonstrating a potential greater stress response in the muscle or less ability to cope with oxidative stress, which occurs during postmortem pH decline and sorbitol accumulation. Additionally, a greater abundance of glutathione S-transferase Mu 1 (*P = *0.10) and glutathione S-transferase (*P = *0.08) was noted in SUPZN-NO than CON-RAC at 1 h postmortem (Table 7). Glutathione transferases catalyze glutathione conjugation to respective metabolites to detoxify compounds and reduce oxidative stress (Samanta et al., 2014). In previous studies, the relationship between tenderness and pH with these metabolites has varied. A greater abundance of glutathione peroxidases and glutathione reductase in high ultimate pH poultry pectoralis major muscle has been reported. (Beauclercq et al., 2017). Decreased glutathione S-transferase mu 1 was identified in the greater ultimate pH classification of beef LT muscle at 24 h postmortem (Poleti et al., 2018). These differences could be a function of species and the loss of maintained reducing conditions in muscle during postmortem pH decline (Huff Lonergan et al., 2010). This increase in oxidative stress-related proteins could also demonstrate the negative impacts of the polyol pathway and its use of NADPH as a necessary compound for glutathione production.

**Table 7.** Stress response proteins differing between nutritional treatments at 1 h and 1 d postmortem

Protein	Accession number	Gene	Coverage, %	Unique peptides	Log 2-FC^1^	*P*-value	Location	Comparison
1 h								
Glutathione S-transferase	Q2KIV8	GSTM3	61	11	2.55	0.08	Cytosol	SUPZN-NO/CON-RAC
Glutathione S-transferase Mu 1	A1A4L7	GSTM4	50	3	0.72	0.10	Cytosol, extracellular	SUPZN-NO/CON-RAC
1 d								
Peroxiredoxin-3	P35705	PRDX3	17	3	0.26	0.05	Mitochondria, sarcoplasm	CON-RAC/CON-NO
Peroxiredoxin-1	Q5E947	PRDX1	56	10	0.15	0.06	Sarcoplasm	CON-RAC/CON-NO
Peroxiredoxin-3	P35705	PRDX3	17	3	0.30	0.01	Mitochondria	SUPZN-NO/CON-NO
Peroxiredoxin-1	Q5E947	PRDX1	56	10	0.20	0.03	Sarcoplasm	SUPZN-NO/CON-NO
Glutathione S-transferase P	P28801	GSTP1	77	11	0.14	0.06	Sarcoplasm, mitochondria, nucleus	SUPZN-NO/CON-NO
Peroxiredoxin-1	Q5E947	PRDX1	56	10	0.18	0.03	Sarcoplasm	SUPZN-RAC/CON-NO

^1^Log 2-FC indicates protein abundance differences between treatments. Positive values are more abundant in the treatment comparison identified first in the comparison column. Negative values are less abundant in the treatment comparison identified first in the comparison column.

CON = no supplemental Zn (analyzed 36 mg Zn/kg DM); SUPZN = CON + 60 mg Zn/kg DM from ZnSO4 + 60 mg Zn/kg DM from Zn–amino acid complex (Availa-Zn; Zinpro Corporation). Fed for the entire 89 d trial.

NO = no supplemental RH; RH = 300 mg RH per head per d (Actogain45; Zoetis, Parsippany, NJ) starting 28 d prior to harvest.

CON-NO = No Zn (36 mg/kg DM basis) or RH supplementation. CON-RAC = No Zn supplementation, only RH supplementation (300 mg/steer/d for 28 d). SUPZN-NO = Zn supplementation (60 mg Zn/kg DM as ZnSO4 + 60 mg Zn/kg DM as Zn–amino acid), no RH supplementation. SUPZN-RAC = Zn and RH supplementation.

Peroxiredoxins are a family of proteins that relieve oxidative stress (Patterson et al., 2021) Due to the use of the polyol pathway and potential impacts on glutathione production, greater peroxiredoxins may have been needed to alleviate the oxidative stress induced by the polyol pathway. A greater abundance of peroxiredoxin-1 and -3 was identified in SUPZN-NO than CON-NO at 1 d postmortem (Table 7; *P = *0.03 and *P = *0.01, respectively). Peroxiredoxins are peroxidases in cells that protect cells against oxidative stress by reducing peroxides with their conserved cysteine residues (Rhee et al., 2001) and are linked to variations in meat quality (Johnson et al., 2023c). Peroxiredoxin-1 and -3 are 2-cysteine containing peroxiredoxins with peroxiredoxin-1 found in the cytosol and peroxiredoxin-3 found primarily in the mitochondria (Heo et al., 2020). Accumulation of hyperoxidized peroxiredoxin-3 is suggested to induce apoptosis (Mun et al., 2020). In peroxiredoxin-3 knockout mice, mitochondrial dysfunction occurs, showing an important biological role of peroxiredoxin-3 on mitochondria homeostasis (Lee et al., 2014). Identifying peroxiredoxin-3 in the sarcoplasmic fraction of muscle could indicate mitochondrial disruption and thus its release into this water-soluble fraction.

A negative relationship between peroxiredoxin-1 and tenderness values has been shown in the sarcoplasmic proteome of Nellore cattle classified by tenderness value (moderately tender: WBSF = 3.9 kg; moderately tough: WBSF = 5.6 kg; very tough: WBSF = 7.9 kg) of the LT muscle at 1 d postmortem (Malheiros et al., 2021). Peroxiredoxin-6 abundance was negatively correlated with beef LT steak sensory tenderness (Zhu et al., 2023). The current study demonstrates a positive relationship between tenderness and peroxiredoxin-1 in the SUPZN-NO treatment at 1 d postmortem compared to CON-NO (Table 7). This difference in the relationship could be explained by breed differences between studies and feeding treatments, as they could influence the proteome of postmortem muscle. Oxidative stress in postmortem muscle can profoundly impact metabolic, structural, and stress-related proteins linked to the development of beef tenderness (Malheiros et al., 2019). Interestingly, in the comparison of CON-RAC to CON-NO, CON-RAC had a greater abundance of peroxiredoxin-1 and -3 at 1 d postmortem (Table 7; *P = *0.06 and *P = *0.05, respectively). The SUPZN-RAC treatment also had a greater abundance of peroxiredoxin-1 than CON-NO at 1 d postmortem. Overall, the CON-NO treatment had less peroxiredoxin’s in the sarcoplasmic protein fraction compared to the sarcoplasmic protein fraction from cattle-fed Zn, RH, or Zn and RH in combination (Table 7). Further investigation into these impacts is necessary to fully define the role of stress-related proteins in postmortem muscle and meat. Greater abundance of peroxiredoxin-1 could be an indicator of the cell functioning to alleviate oxidative stress induced by the polyol pathway in the SUPZN-NO treatment. Although this impact in SUPZN-NO could be attributed to the polyol pathway and a more rapid pH decline, this same result in CON-RAC may be due to other factors that are not fully understood and need deeper investigation.

Myosin regulatory light chain 2 (MYL2) was less abundant in SUPZN-NO than CON-NO, CON-RAC, and SUPZN-RAC (Table 4; *P = *0.09, *P = *0.07, and *P = *0.04, respectively). Myosin regulatory light chain 2 fine-tunes myosin’s motor function and ATPase activity for contraction (Clark et al., 2002). Phosphorylation of MYL is necessary for calcium binding and the modulation of myosin’s ATPase cycle (Sweeney and Hammers, 2018). In vitro dephosphorylation of MYL has shown a negative influence on actomyosin dissociation and a positive impact on the interacting force between actin and myosin (Gao et al., 2017). In a study investigating the impacts of phosphorylation on MYL related to actomyosin dissociation and myosin degradation, phosphorylation levels of MYL decreased from 0.5 to 72 h postmortem (Cao et al., 2019). Cao et al. (2019) showed that degradation of MYL and MHC increased with decreased phosphorylation. As the sarcoplasmic protein fraction of muscle from cattle-fed Zn-only had a less abundance of MYL2 than all other treatments at 1 h postmortem (Table 4), Zn-only could be impacting the solubility of MYL2 in early postmortem muscle as this was identified in the sarcoplasmic protein fraction. This solubility of MYL2 could be affecting rigor development in postmortem muscle as the muscle from Zn-only fed cattle had a more rapid pH decline and lower WBSF value at 1 d postmortem (Table 1).

Zeta-crystallin was greater in abundance in the sarcoplasmic protein fraction of muscle from SUPZN-NO than all other treatments at 1 h postmortem (Table 3; *P* ≤ 0.04). This protein was first identified as a DNA binding protein and found in low abundance in most tissues (Lulli et al., 2020). More recently, roles for this protein in phosphorylation (Lee et al., 2011) and apoptosis (Lulli et al., 2020) have been identified. Zn has also been identified to impact apoptotic functions in a dose-dependent manner with greater zinc concentrations inducing apoptosis (Franklin and Costello, 2009; Kocdor et al., 2015). Zeta-crystallin has been suggested to regulate anti-apoptotic factors and thus could play a regulatory role in the apoptotic process in postmortem muscle (Lulli et al., 2020). The Zn-only treatment (SUPZN-NO) could have greater zeta-crystallin due to differences in postmortem pH decline that is causing apoptotic activation earlier postmortem. This effect due to Zn-only supplementation needs to be investigated.

The SUPZN-NO treatment had a greater abundance of calcium-binding proteins in the sarcoplasmic fraction (Cadherin-13 [*P = *0.01], EH-domain containing protein 2 [*P = *0.02], serine/threonine-protein phosphatase [*P = *0.07], annexin A3 [*P = *0.09], and kininogen-1 [*P = *0.07]) than CON-NO at 1 h postmortem (Table 8). More calcium-binding proteins (annexin A5 [*P = *0.05], protein S100-A1 [*P = *0.09], and peptidyl–prolyl cis-trans isomerase FKBP1A [*P = *0.02]) were found in SUPZN-NO than CON-RAC at 1 h postmortem (Table 8). These proteins can bind calcium ions and are found in cell membranes, the mitochondria, plasma membrane, nucleus, sarcoplasmic reticulum, and extracellular. These proteins could influence postmortem protein degradation. For example, kinonogen-1 has been shown to inhibit calpain activity (Schmaier et al., 1986). It is proposed that the presence of these proteins in the sarcoplasmic fraction indicates disruption of cellular and subcellular membranes. Thus, a greater abundance of these phosphorylated proteins in the sarcoplasmic fraction indicates protein degradation and cellular disorder. Muscle disruption occurs due to protease activity and structural changes in postmortem muscle (Kim et al., 2018), explaining why these proteins were found in greater abundance in the sarcoplasmic fraction of the SUPZN-NO treatment. However, a greater abundance of these calcium-binding proteins in these organelles in the SUPZN-NO treatment could exist. Further investigation to identify if greater disruption may have occurred or if a greater abundance of calcium-binding proteins exists in SUPZN-NO is needed.

**Table 8.** Calcium-binding proteins differing between nutritional treatments at 1 h postmortem

Protein	Accession number	Gene	Coverage, %	Unique peptides	Log 2-FC^1^	*P*-value	Location	Comparison
Cadherin-13	Q3B7N0	CDH13	4	2	1.50	0.01	Cell membrane	SUPZN-NO/CON-NO
EH-domain containing 2	Q2KJ47	EHD2	3	1	0.76	0.02	Membrane	SUPZN-NO/CON-NO
Serine/threonine-protein phosphatase	A0A3Q1M4S3	PPP3CA	9	2	0.28	0.07	Nucleus, mitochondria, cytosol	SUPZN-NO/CON-NO
Kininogen-1	A0A140T8C8	KNG1	1	1	1.00	0.07	Extracellular	SUPZN-NO/CON-NO
Annexin A3	F1MWQ2	ANXA3	5	1	0.58	0.09	Plasma membrane, sarcoplasm	SUPZN-NO/CON-NO
Annexin A5	A1L5B6	ANXA5	46	1	0.20	0.05	Nuclear membrane	SUPZN-NO/CON-RAC
Protein S100-A1	P02639	S100A1	16	1	0.83	0.09	Sarcoplasm, mitochondria, Sarcoplasmic reticulum	SUPZN-NO/CON-RAC
Peptidyl–prolyl cis-trans isomerase FKBP1A	P18203	FKBP1A	56	4	0.13	0.02	Sarcoplasmic reticulum, cytosol	SUPZN-NO/CON-RAC

^1^Log 2-FC indicates protein abundance differences between treatments. Positive values are more abundant in the treatment comparison identified first in the comparison column. Negative values are less abundant in the treatment comparison identified first in the comparison column.

CON = no supplemental Zn (analyzed 36 mg Zn/kg DM); SUPZN = CON + 60 mg Zn/kg DM from ZnSO4 + 60 mg Zn/kg DM from Zn–amino acid complex (Availa-Zn; Zinpro Corporation). Fed for the entire 89 d trial.

NO = no supplemental RH; RH = 300 mg RH per head per d (Actogain45; Zoetis, Parsippany, NJ) starting 28 d prior to harvest.

CON-NO = No Zn (36 mg/kg DM basis) or RH supplementation. CON-RAC = No Zn supplementation, only RH supplementation (300 mg/steer/d for 28 d). SUPZN-NO = Zn supplementation (60 mg Zn/kg DM as ZnSO4 + 60 mg Zn/kg DM as Zn–amino acid), no RH supplementation. SUPZN-RAC = Zn and RH supplementation.

Muscle from supranutritional Zn-only treatment (SUPZN-NO) had less HSPA4 than all other treatments in the phosphoproteome analysis (Table 6; Fig. 1; *P* ≤ 0.01). Phosphoproteome analysis also showed greater isoforms of heat shock 70-kDa protein 1A (HSPA1A; 2 spots) in SUPZN-NO than CON-RAC and less HSPA1A (2 spots) in SUPZN-RAC than CON-NO and SUPZN-NO in the phosphoproteome analysis (Table 6; Fig. 1). Heat shock proteins typically are thought to negatively affect meat tenderness due to their protective role against cellular stress, structural homeostasis, and apoptosis; and thus, cell survival in postmortem muscle (Picard and Gagaoua, 2020a, b). In the majority of tenderness studies, a lesser abundance of HSPA1A has been identified in more tender meat (Bjarnadóttir et al., 2012; Carvalho et al., 2014; Poleti et al., 2018; Malheiros et al., 2021), with one identified study showing a positive relationship with tenderness (D’Alessandro et al., 2012), however, it must be noted that these studies analyzed the proteome of muscle, not phosphoproteome. Phosphorylation of heat shock proteins can impact their ability to be degraded, thus influencing their role in protecting cells and structural proteins. For example, heat shock protein beta-1 degradation occurred more quickly in the muscle that had inhibited protein kinase activity (Li et al., 2018). As these impacts on heat shock proteins were seen in multiple comparisons of Zn treatments, the potential role of phosphorylation and Zn as a modulator of phosphorylation is necessary to understand this difference and its influence on tenderness development.

### Proteome and metabolome analysis: CON-RAC and SUPZN-RAC

At 1 h postmortem, CON-RAC had a less abundance of amino acids (isoleucine, phenylalanine, glycine, and alanine; *P* ≤ 0.08), and SUPZN-RAC had a less alanine (*P = *0.03) compared to CON-NO (Table 9). At 1 h postmortem, differences in protein degradation resulting in the release of peptides and individual amino acids are not expected. This RH effect of less amino acids at 1 h postmortem could relate to the protein accretion exhibited in cattle-fed RH (Mersmann, 1998). Feeding RH during the final feeding phase of production results in increased lipolysis and decreased protein degradation in muscle causing greater protein accretion instead of lipid accretion. This shift to increased protein accretion could impact amino acid use and influence the less abundance of amino acids at 1 h postmortem in cattle-fed RH due to the need for amino acids for protein accumulation.

**Table 9.** Effect of supranutritional zinc (Zn) and RH supplementation on the beef LT muscle metabolome at 1 h postmortem

Pathway	Metabolite	Comparison^1^^,^^2^	Log 2-FC^3^	*P*-value
Other	TrisCarbamate	CON-RAC/CON-NO	−0.41	0.01
Nitrogen metabolism	Urea	CON-RAC/CON-NO	−0.46	0.04
Other	Erythronic acid	CON-RAC/CON-NO	−1.46	0.05
Amino acid	Isoleucine	CON-RAC/CON-NO	−0.43	0.05
Amino acid	Phenylalanine	CON-RAC/CON-NO	−0.48	0.05
Amino acid	Glycine	CON-RAC/CON-NO	−0.35	0.07
Amino acid	Alanine	CON-RAC/CON-NO	−0.46	0.08
Other	Hydroxybutyric acid	SUPZN-NO/CON-NO	1.14	0.06
Amino acid	Alanine	SUPZN-RAC/CON-NO	−0.57	0.03
Amino acid	Isoleucine	SUPZN-NO/CON-RAC	0.57	0.04
Fatty acid	Linoleic acid	SUPZN-NO/CON-RAC	−0.84	0.06
Amino acid	Oxoproline	SUPZN-NO/CON-RAC	−0.64	0.09
Fatty acid	Linoleic acid	SUPZN-RAC/CON-RAC	−0.69	0.02
TCA cycle	Malate	SUPZN-RAC/SUPZN-NO	−0.63	0.08
Other	Hydroxylamine	SUPZN-RAC/SUPZN-NO	0.82	0.10

^1^CON = no supplemental Zn (analyzed 36 mg Zn/kg DM); SUPZN = CON + 60 mg Zn/kg DM from ZnSO4 + 60 mg Zn/kg DM from Zn–amino acid complex (Availa-Zn; Zinpro Corporation). Fed for the entire 89 d trial.

^2^NO = no supplemental RH; RH = 300 mg RH per head per d (Actogain45; Zoetis, Parsippany, NJ) starting 28 d prior to harvest.

^3^Log 2-FC indicates metabolite abundance differences between treatments. Positive values are more abundant in the treatment comparison identified first in the comparison column. Negative values are less abundant in the treatment comparison identified first in the comparison column.

CON-NO = No Zn (36 mg/kg DM basis) or RH supplementation. CON-RAC = No Zn supplementation, only RH supplementation (300 mg/steer/d for 28 d). SUPZN-NO = Zn supplementation (60 mg Zn/kg DM as ZnSO4 + 60 mg Zn/kg DM as Zn–amino acid), no RH supplementation. SUPZN-RAC = Zn and RH supplementation.

Myoglobin and several structural proteins differed in abundance in the sarcoplasmic protein fraction. CON-RAC had greater actin, troponin-T, tropomyosin alpha-1 chain, and myosin-7 (Table 4; *P* ≤ 0.10) and less myoglobin (Table 3; *P = *0.09) than CON-NO at 1 h postmortem. Interestingly, the combination treatment, SUPZN-RAC, also had a greater abundance of structural proteins (myosin light chain kinase 2, myosin-7, MHC 1, MHC 4, and troponin-T) than CON-NO at 1 h postmortem (Table 4; *P* ≤ 0.09). SUPZN-RAC also had a greater abundance of MYL2, troponin C, tropomyosin alpha-3 chain, myosin light polypeptide 6, actin, and desmin than SUPZN-NO at 1 h postmortem (Table 4*P* ≤ 0.09). Overall, a greater abundance of structural proteins in the sarcoplasmic protein fraction at 1 h postmortem in the muscle of cattle-fed RH was found. A greater abundance of structural proteins could be related to several things. First, this could suggest fiber type shifts even though the MHC analysis was not different. These proteins could identify shifts in type II fibers (IIa and IIx) or hypertrophy of fiber types, as the current studies analysis method only measured type I and type II fibers. Another explanation is that these structural proteins could result from some protein degradation, but this greater abundance of structural proteins in RH-fed cattle muscle was identified at 1 h postmortem. Great amounts of protein degradation would not be expected at 1 h postmortem, however, with the use of new proteomic methods (TMT labeling), detection of these peptide level differences may be possible compared to previous gel-based methods.

Deeper investigation into structural proteins is necessary, such as actin, as actin has been demonstrated to differ in abundance in breeds with known differences in muscle metabolism (D’Alessandro et al., 2011; Lana and Zolla, 2016). Muscle from cattle-fed the CON-RAC treatment at 1 h postmortem had a lesser abundance of proteins that have functions to bind and influence actin integrity (Pollard, 2016). These proteins include gelsolin, protein FAM49B, and vitamin D-binding protein compared with the CON-NO treatment (Table 10). For example, gelsolin is an actin-binding protein that depolymerizes actin and can function with vitamin D-binding protein to form a scavenger system that can depolymerize actin filaments (Pollard, 2016). Muscle from CON-RAC had less serotransferrin, T-complex protein 1 subunit theta, and WD repeat-containing protein 1 at 1 h postmortem compared with SUPZN-NO (Table 10; *P* ≤ 0.09). Lastly, less serotransferrin and smoothelin like 1 were identified in SUPZN-RAC muscle than SUPZN-NO at 1 h postmortem (Table 10; *P* ≤ 0.03). A lesser abundance of proteins related to actin-binding and maintenance of the integrity of actin in muscle from animals supplemented RH could point towards a relationship to the greater abundance of actin found in the sarcoplasmic muscle protein fraction of animals supplemented RH. This consistent result across treatments identifies the need to understand if and how increased actin may be impacting postmortem metabolism or tenderness development.

**Table 10.** Actin-binding proteins differing between nutritional treatments at 1 h postmortem

Protein	Accession number	Gene	Coverage, %	Unique peptides	Log 2-FC^1^	*P*-value	Location	Comparison
Gelsolin	F1N1I6	GSN	4	3	−0.18	0.04	Phagosome, focal adhesion	CON-RAC/CON-NO
Protein FAM49B	A0A3Q1N7H1	CYRIB	7	2	−0.15	0.04	Mitochondria	CON-RAC/CON-NO
Vitamin D-binding protein	A0A3Q1LQ02	GC	14	6	−0.20	0.06	Secreted	CON-RAC/CON-NO
Serotransferrin	G3X6N3	TF	58	38	0.14	0.06	Secreted	SUPZN-NO/CON-RAC
T-complex protein 1 subunit beta	Q3ZBH0	CCT2	6	2	1.27	0.09	Sarcoplasm	SUPZN-NO/CON-RAC
WD repeat-containing protein 1	F1MTP5	WDR1	18	9	0.37	0.09	Cytoskeleton, membrane	SUPZN-NO/CON-RAC
Smoothelin like 1	E1BPV6	SMTNL1	31	9	−0.79	0.06	Sarcomere, nucleus	SUPZN-RAC/SUPZN-NO
Serotransferrin	G3X6N3	TF	58	38	−0.18	0.07	Secreted	SUPZN-RAC/SUPZN-NO

^1^Log 2-FC indicates protein abundance differences between treatments. Positive values are more abundant in the treatment comparison identified first in the comparison column. Negative values are less abundant in the treatment comparison identified first in the comparison column.

CON = no supplemental Zn (analyzed 36 mg Zn/kg DM); SUPZN = CON + 60 mg Zn/kg DM from ZnSO4 + 60 mg Zn/kg DM from Zn–amino acid complex (Availa-Zn; Zinpro Corporation). Fed for the entire 89 d trial.

NO = no supplemental RH; RH = 300 mg RH per head per d (Actogain45; Zoetis, Parsippany, NJ) starting 28 d prior to harvest.

CON-NO = No Zn (36 mg/kg DM basis) or RH supplementation. CON-RAC = No Zn supplementation, only RH supplementation (300 mg/steer/d for 28 d). SUPZN-NO = Zn supplementation (60 mg Zn/kg DM as ZnSO4 + 60 mg Zn/kg DM as Zn–amino acid), no RH supplementation. SUPZN-RAC = Zn and RH supplementation.

Postmortem metabolic rates and pH decline were different between treatments. Differences in metabolite and protein abundances could help explain energy pathways utilized within muscle from each treatment. The most rapid pH decline, SUPZN-NO, potentially utilized the polyol pathway resulting in less ATP production and a more rapid accumulation of lactate and hydrogen ions. At 1 d postmortem, metabolite analysis of muscle from cattle-fed RAC-only (CON-RAC) had a lesser abundance of glucose, glucose-6-phosphate, and glycine than CON-NO and SUPZN-NO (Table 2; *P* ≤ 0.08). At 1 h postmortem, TMT analysis of muscle from the CON-RAC treatment had a lesser abundance of glyceraldehyde-3-phosphate dehydrogenase than CON-NO and SUPZN-RAC (Table 5; *P = *0.08). Lastly, in the CON-RAC treatment, muscle phosphoproteome analysis revealed a greater abundance of 4-trimethylaminobutyraldehyde dehydrogenase (ALDH9A1), ATP synthase F1 subunit beta (ATP5F1B), and malate dehydrogenase (MDH1) compared to all other treatments (Table 6; Fig. 1; *P* ≤ 0.10). As the Zn-only treatment may have utilized the polyol pathway to clear excess glucose conditions, the CON-RAC treatment may have dealt with these conditions through glycolytic and oxidative metabolism mechanisms discussed below. These differences in metabolites and proteins may shed light on the molecular mechanisms to understand the postmortem pH decline differences between Zn and RH supplemented animals.

ATP5F1B and MDH1 are found in the mitochondria, potentially demonstrating mitochondrial disruption in the CON-RAC treatment. MDH1 plays a role in the citric acid cycle to convert malate to oxaloacetate. MDH1 has been identified as a potential biomarker of tenderness, most likely due to its involvement in energy metabolism and postmortem pH decline (Bonnet et al., 2020). MDH1 abundance in Blond d ’Aquitaine was negatively correlated with pH at 45 min postmortem in the LT muscle at 30 min postmortem (Gagaoua et al., 2015). Similarly, MDH1 was less abundant in early postmortem beef that demonstrated an ultimate high pH (Patinho et al., 2024). A positive relationship between MDH1 abundance and tenderness is suggested (Bonnet et al., 2020); however, an investigation into the influence of phosphorylation on MDH1 in the soluble protein fraction is necessary to understand the influence of pH decline and tenderness development.

In the phosphoproteome analysis at 1 h postmortem of the current study, the samples with the least rapid pH decline (CON-RAC) had a greater abundance of ATP5F1B than CON-NO, SUPZN-NO, and SUPZN-RAC (Table 6; Fig. 1). ATP5F1B is involved in the electron transport chain for energy production (Stock et al., 1999). The beta subunit of ATP synthase is water-soluble and can play a role in extending pH decline (Matarneh et al., 2018). The phosphorylated isoform of ATP5F1B was down-regulated in diabetic human muscle, demonstrating influences on energy metabolism (Højlund et al., 2003). Phosphorylation of the beta subunit of ATP synthase can influence the structure and function of the F_1_F_0_ ATP synthase complex (Kane et al., 2010). In Chinese Luxi yellow cattle longissimus lumborum muscle, ATP5F1B abundance at 1 d postmortem in the sarcoplasmic fraction was negatively (−0.95) correlated with pH at 1 d postmortem (Wu et al., 2016). A greater understanding of the impacts of ATP5F1B and the influence of phosphorylation is necessary to understand how it may be influencing CON-RAC, the slowest declining samples.

## Conclusions

As hypothesized, differences in the metabolome, proteome, and phosphoproteome were identified in cattle LT muscle that varied in pH decline and tenderness development. Pathways related to energy metabolism, stress response, and structural proteins and metabolites differed between treatments. In SUPZN-NO, increased sorbitol and fructose concentrations were observed and suggested use of the polyol pathway due to increased glucose concentrations. In samples with the slowest pH decline and greatest WBSF value at 1 d postmortem (CON-RAC), differences in energy metabolism enzymes (abundance and phosphorylation status) could explain the slower pH decline. Specifically, the abundance of PGM1, ATP5F1B, and malate dehydrogenase spots identified in the phosphoproteome analysis could extend pH decline in the CON-RAC treatment, while the use of the polyol pathway and decreased AMPD1 in SUPZN-NO treatment could impact ATP production and result in more rapid lactate buildup resulting in a lower pH. Greater stress response proteins at 1 d postmortem were identified in SUPZN-NO and could be due to the use of the polyol pathway and removal of NADPH which impacts the oxidative status of muscle. Less MYL2 in SUPZN-NO at 1 h postmortem could signify greater actomyosin dissociation and partially explain the reduced WBSF value at 1 d postmortem.

Additionally, a greater abundance of structural proteins (troponin-T, myosin-7, and actin) was found in the muscle proteome from animals supplemented RH (CON-RAC and SUPNZN-RAC) than CON-NO and SUPZN-NO at 1 h postmortem. These structural proteins could influence rigor development and thus potentially impact pH decline and tenderness development differences observed at 1 d postmortem. Overall, nutritional supplementation influenced differences in postmortem metabolism pathways which impacted pH decline and resulted in WBSF differences influenced by structural protein abundances and phosphorylation status.

## Abbreviations

AMPD: adenosine monophosphate deaminase
AMPD1: adenosine monophosphate deaminase 1
AMPER: ammonium persulfate
ATP: adenosine triphosphate
ATP5F1B: ATP synthase F1 subunit beta
DM: dry matter
DTT: dithiothreitol
EDTA: ethylenediaminetetraacetic acid
FC: fold change
FDR: false discovery rate
GC-MS: gas chromatography-mass spectrometry
HSPA1A: heat shock 70-kDa protein 1A
LT: longissimus thoracis
MDH1: malate dehydrogenase
MHC: myosin heavy chain
MYL2: myosin regulatory light chain 2
NADPH: nicotinamide adenine dinucleotide phosphate
PGM1: phosphoglucomutase-1
RH: ractopamine Hydrochloride
SDS: sodium dodecyl sulfate
TEMED: tetramethylene diamine
TMT: tandem mass tagging
WBSF: Warner–Bratzler shear force
Zn: zinc
